# Cardiac manifestations of human *ACTA2* variants recapitulated in a zebrafish model

**DOI:** 10.1038/s10038-024-01221-0

**Published:** 2024-02-05

**Authors:** Wulan Apridita Sebastian, Masanori Inoue, Nobuyuki Shimizu, Ryosuke Sato, Saori Oguri, Tomoyo Itonaga, Shintaro Kishimoto, Hiroshi Shiraishi, Toshikatsu Hanada, Kenji Ihara

**Affiliations:** 1 Department of Pediatrics, Oita University, Faculty of Medicine, Oita, Japan; 2 Department of Cell Biology, Oita University, Faculty of Medicine, Oita, Japan

**Keywords:** Experimental models of disease, Genetics, Congenital heart defects

## Abstract

The *ACTA2* gene encodes actin α2, a major smooth muscle protein in vascular smooth muscle cells. Missense variants in the *ACTA2* gene can cause inherited thoracic aortic diseases with characteristic symptoms, such as dysfunction of smooth muscle cells in the lungs, brain vessels, intestines, pupils, bladder, or heart. We identified a heterozygous missense variant of Gly148Arg (G148R) in a patient with a thoracic aortic aneurysm, dissection, and left ventricular non-compaction. We used zebrafish as an in vivo model to investigate whether or not the variants might cause functional or histopathological abnormalities in the heart. Following the fertilization of one-cell stage embryos, we injected in vitro synthesized *ACTA2* mRNA of wild-type, novel variant G148R, or the previously known pathogenic variant Arg179His (R179H). The embryos were maintained and raised for 72 h post-fertilization for a heart analysis. Shortening fractions of heart were significantly reduced in both pathogenic variants. A histopathological evaluation showed that the myocardial wall of *ACTA2* pathogenic variants was thinner than that of the wild type, and the total cell number within the myocardium was markedly decreased in all zebrafish with pathogenic variants mRNAs. Proliferating cell numbers were also significantly decreased in the endothelial and myocardial regions of zebrafish with *ACTA2* variants compared to the wild type. These results demonstrate the effects of *ACTA2* G148R and R179H on the development of left ventricle non-compaction and cardiac morphological abnormalities. Our study highlights the previously unknown significance of the *ACTA2* gene in several aspects of cardiovascular development.

## Introduction

Inherited aortic aneurysms and dissections are life-threatening genetic syndromes [1]. They are classified into two categories: syndromic arterial aneurysms, which display phenotypes across multiple systems other than the aorta; and non-syndromic arterial aneurysms, which have less extensive phenotypes. Syndromic aortic aneurysms, such as Marfan syndrome and Loeys-Dietz syndrome, often exhibit a high penetrance of structural and functional abnormalities in the connective tissue [1]. In addition to aortic root enlargement, Marfan syndrome also exhibits skeletal features, such as transposition of the lens, wrist and thumb signs, chest wall deformity, hind foot deformity, scoliosis, pneumothorax, dural ectasia, and acetabular protrusion [2]. In Loeys-Dietz syndrome, distinctive clinical features appear in the skeletal system without ectopia lentis [3]. Vascular Ehlers-Danlos syndrome is characterized by serious complications in connective tissues, such as recurrent formation and dissection of aneurysms in the aorta or intestinal arteries, uterine and intestinal rupture, or pneumothorax [4].

In contrast, non-symptomatic aneurysms often have abnormalities in genes specifically expressed in vascular component cells, such as vascular smooth muscle cells (SMCs). Genetic anomalies of the genes encoding smooth muscle contraction units (ACTA2, MYH11, MYLK, and PRKG1) have been recognized [5]. Unlike syndromic arterial aneurysms, it is difficult to make a diagnosis from external signs, and many patients are diagnosed with underlying genetic abnormalities after the onset of severe aortic dissection. Consequently, it is crucial to detect specific findings among non-specific findings and diagnose them before the onset of severe vascular sequelae, such as arterial dissection.

The *ACTA2* gene, which encodes the vascular SMC-specific isoform of α-actin, is a major component of the contractile apparatus in SMCs located in the arterial system [6]. The pathogenic variant of the ACTA2 gene accounts for 14% of inherited ascending thoracic aortic aneurysms and dissections [7]. It also causes various vascular disorders in the coronary arteries and intracranial arteries. SMCs containing aberrant ACTA2 fibers are susceptible to disruption of the actin fiber assembly or stability. Specific variants in the *ACTA2* gene, such as Arg179His (R179H), cause multisystemic smooth muscle dysfunction syndrome (MSDS), which is associated with additional vascular complications, such as congenital mydriasis, chronic interstitial lung disease, hypoperistalsis, hydrops of the gall bladder or hypotonic bladder, aneurysmal dilatations and dissections, patent ductus arteriosus, and early-onset coronary artery disease [5, 7, 8].

In addition to its function in vascular SMCs, *ACTA2* is essential for proper cardiovascular organ development [6]. During the early developmental stage, ACTA2 expression occurs in parallel with the appearance of beating areas, indicating the formation of cardiomyocytes [9]. Genetic abnormalities in the *ACTA2* gene can thus cause congenital heart disease.

We herein report a patient with a heterozygous variant of Gly148Arg (G148R) in the *ACTA2* gene demonstrating MSDS with a rare manifestation of left ventricular non-compaction (LVNC). This variant has been reported only in a single family [5]. To confirm the pathogenicity of the rare variant in the cardiac development and function, we developed a zebrafish model mimicking the genetic disorder caused by an *ACTA2* mutation and evaluated its pathophysiological effects on the affected heart.

## Material and methods

### Clinical evaluations of family members

The clinical evaluation of the patient was performed at the Department of Pediatrics, Oita University Hospital, Oita, Japan, with their written informed consent, in accordance with the local human ethics standards (Clinical ethic number 2624). The evaluation included detailed personal and family histories, a physical examination, a 12-lead electrocardiogram (ECG) recording, echocardiography, cardiac or abdominal magnetic resonance imaging (MRI), 24-h Holter monitoring, and a review of the medical records.

### Zebrafish husbandry

All zebrafish (AB strain and Tg[*cmlc2*:EGFP] line) were raised and maintained according to standard procedures. They were kept under a 14-h-light:10-h-dark cycle at 28-29 °C. Embryos were harvested and placed at 28.5 °C. All experimental animal procedures were performed in accordance with the institutional and national guidelines.

The study was conducted in compliance with the ARRIVE guidelines. The study protocol was approved by the Institutional Review Board of Oita University (approval numbers 230501 and 4-5).

### mRNA microinjection in zebrafish and a heart contraction analysis

Human *ACTA2* mRNA was generated by in vitro transcription using the mMESSAGE mMACHINE T7 transcription kit (AM1344; Invitrogen, Carlsbad, CA, USA), according to the manufacturer’s protocol. Fertilized one-cell stage Tg[*cmlc2*:EGFP] transgenic line embryos were injected with 50 pg human *ACTA2* mRNA variant (WT, G148R, and R179H). In vivo videos of the beating heart in Tg[*cmlc2*:EGFP] larvae with *ACTA2* mRNA at 3 days post fertilization (dpf) were obtained using a fluorescent stereo microscope (BZ-9000; Keyence, Osaka, Japan). The heart rate was measured, and cardiac contractility was assessed by analyzing the dynamic changes in the intensity of the EGFP fluorescence signal [10, 11]. Videos of the heart region were recorded as follows: the heartbeats were documented in 30-sec videos and imported into the ImageJ software program (Bethesda, MD, USA; https://imagej.nih.gov/ij/). The heart region was outlined using the EGFP signal to compare its size. Signal intensities were obtained by selecting the same region of interest (ROI) in the ventricular regions using the circle tool. The resulting plot profiles were analyzed to determine the cardiac rhythm for each image in the stack. Shortening fractions (SF) were calculated using the following formula: SF = (length at diastole – length at systole) / length at diastole × 100 [11]. The heart rate was obtained by dividing the total number of heartbeats by the video length.

### Immunofluorescence staining

For cryosections, 4 dpf larvae were fixed with 4% paraformaldehyde for 16-h and then incubated in a microcentrifuge tube with 30% sucrose in phosphate-buffered saline until the samples sank to the bottom of the tube. All samples were transversally embedded in a mixture of 30% sucrose and Tissue-Tek O.C.T compound (4583; Sakura-Finetek, Tokyo, Japan; 2:1) and fixed in liquid nitrogen. Serial sections were obtained using a Leica CM1950 microtome. An immunofluorescence analysis was performed using monoclonal anti-proliferating cell nuclear antigen (PCNA) (P8825; Sigma-Aldrich, St.Louis, MO, USA; 1:200) as the primary antibody. Alexa Fluor 555 donkey anti-mouse IgG (A21206; Molecular Probes, Eugene, OR, USA; 1:500) was used as the secondary antibody. To confirm the endogenous expression of ACTA2 protein in the heart, immunofluorescence analysis was performed using anti-acta2 (GTX124505, GeneTex Inc, North America, 1:100) as primary antibody and Alexa Fluor 555 goat anti-rabbit IgG (A21428; Molecular Probes, Eugene, OR, USA; 1:500) as the secondary antibody. Images were captured using a laser scanning microscope (BZ-X800; Keyence).

### Statistical analyses

All values are presented as mean ± standard deviation. Comparisons between groups were made by one-way ANOVA followed by post hoc test. Statistical significance was set at *P* < 0.05. Statistical analyses were performed using the GraphPad Prism software program, version 9 (GraphPad Software Inc., San Diego, CA, USA).

## Results

### Clinical report

The proband was a 14-year-old Japanese boy who had been born at 38 weeks’ gestation weighing 2600 *g* to non-consanguineous parents. The patient had bronchial asthma since early childhood. At six years old, he underwent orchiopexy for cryptorchidism. His family history revealed that his 44-year-old father had urolithiasis, and his 38-year-old mother had hypertension. The patient’s maternal grandfather had hypertension, diabetes, and pancreatic cancer. Both his maternal uncle and aunt had bronchial asthma. His 21-year-old brother was healthy.

**Fig. 1:**
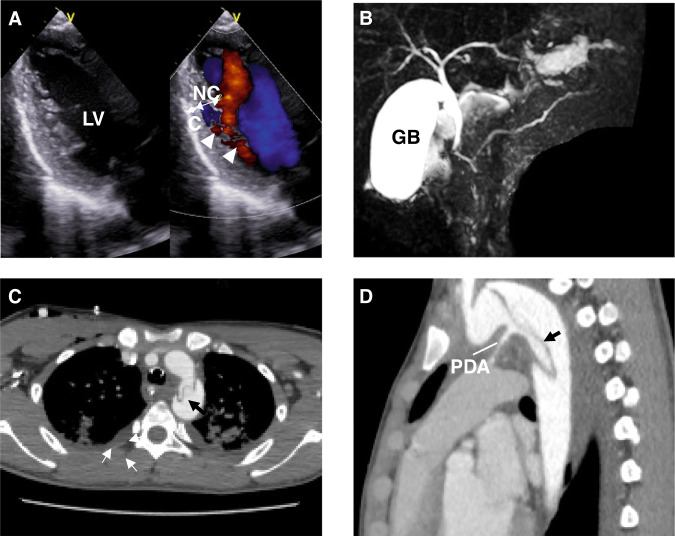
Echocardiography and magnetic resonance cholangio-pancreatography (MCRP) of the patient with the *ACTA2* G148R variant. **A** An echocardiogram showing the left ventricular long-axis view during diastole. The ventricular wall of the left ventricle exhibits an outer compacted layer (C) and an inner non-compacted trabecular layer (NC), with deep recesses formed by the trabeculations (NC/C ratio 2.0). Color Doppler imaging shows blood infiltrating into the spaces between the trabeculae (arrowheads). **B** On MRCP, the main pancreatic duct exhibits a reverse Z-shape (arrows) in the head of the pancreas region. **C**, **D** Chest magnetic resonance imaging shows the descending aortic deviation. LV left ventricle, C compacted layer, NC non-compacted layer, GB gallbladder, PDA patency of the ductus arteriosus

The patient had had episodes of recurrent vomiting and abdominal pain since he was 10 years old. An ultrasound examination by a physician revealed an acute angled superior mesenteric artery (SMA) originating from the aorta and dilation of the descending part of the duodenum, leading to a diagnosis of SMA syndrome. Gallstones were also observed. Despite treatment for SMA syndrome, the frequency of vomiting and abdominal pain did not change, and he was referred to our hospital at 14 years old. His blood pressure was mildly elevated at approximately 130/70 mmHg, and echocardiography revealed the presence of LVNC associated with patent ductus arteriosus (Fig. 1A). At 15 years old, he consulted our department with epigastric pain and vomiting. Plain computed tomography (CT) of the abdomen revealed enlargement of the tail of the pancreas surrounded by increased fat tissue, gallbladder enlargement, gallstones, bladder stones, and kidney stones. Blood tests revealed an elevated pancreatic amylase level (386 U/L). He was diagnosed with acute pancreatitis and admitted to our department. The patient was managed with fasting, massive fluid resuscitation, antibiotics, gabexate mesilate, and a protease inhibitor, which led to improvement. After recovery, abdominal MRI during hospitalization demonstrated a tortuous main pancreatic duct in the shape of a reverse Z (Fig. 1B). Ophthalmic examination revealed dilated pupil. At 16 years old, he was transported to our hospital complaining of epigastric pain. He was in severe distress and sweating, and showed a discrepancy in blood pressure between the upper and lower extremities along with a weak pulse in the left femoral artery. Chest MRI revealed aortic dissection extending from just below the left subclavian artery branch to just below the left common iliac artery branch, leading to a diagnosis of Stanford type B acute aortic dissection (Fig. 1C, D).

The patient underwent right axillo-bilateral femoral artery bypass surgery; however, he developed rhabdomyolysis due to reperfusion injury and experienced cardiac arrest due to hyperkalemia. Cardiopulmonary resuscitation was performed, and systemic circulation was restored; however, on day 15 of the illness, he developed necrotizing cholecystitis. On day 52 of the illness, he had bowel perforation due to volvulus caused by malrotation. He underwent right hemicolectomy and developed peritonitis and sepsis, leading to disseminated intravascular coagulation. Multiple thrombi were observed in the left ventricle without compaction, and multiple emboli were observed in the liver, kidneys, and spleen. The patient died at 16 years old due to multiple organ failure.

### ACTA2 G148R identified as a candidate cause of TAAD (thoracic aortic aneurysm and dissections) and LVNC (left ventricular non-compaction)

Genetic testing using a hybridization capture-based gene panel identified a heterozygous variant of the *ACTA2* gene (c.442 G > A), generating p.Gly148Arg (G148R). The missense variant G148R was predicted to be pathogenic in silico (PolyPhen2: Probably Damaging with a score of 0.995 [sensitivity: 0.68; specificity: 0.97]), and it affected evolutionarily conserved residues. The amino acid residue G148R in *ACTA2* has previously been reported in only one family with TAAD [5].

### Cardiac dysfunction induced by ACTA2 pathogenic variants in vivo

**Fig. 2:**
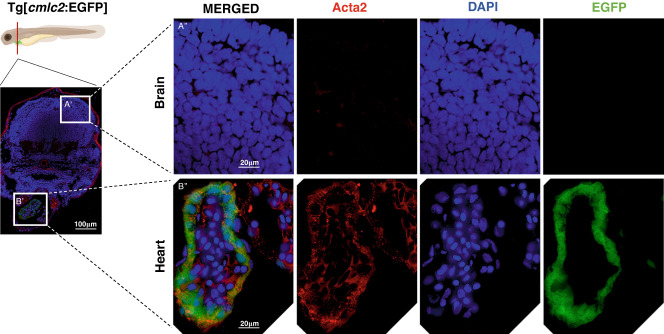
Endogenous *ACTA2* expression in the heart. Schematic figure of transversal section of zebrafish larvae Tg[*cmlc2*:EGFP] at 4 days post-fertilization. Immunostaining of 4dpf larvae Tg[*cmlc2*:EGFP] with anti-acta2 antibody (red), followed by 4’,6-diamidino-2-phenylindole (DAPI) co-staining (blue). **A**’ Brain region. **B**’ Heart region. Scale bar: 100 μm. **A**”, **B**” High magnification of immunostaining image. Scale bar: 20 μm. **A**” In the brain area, endogenous *ACTA2* was not identified. **B**” In the heart area, endogenous *ACTA2* was highly expressed, highlighted with EGFP green fluorescence, anti-acta2 antibody (red), and DAPI. Illustration of zebrafish was created with Biorender.com

First, to verify the endogenous expression of ACTA2 protein in the heart, we conducted immunostaining using the Tg[*cmlc2*:EGFP] transgenic zebrafish line, which expresses the EGFP fluorescent protein specifically in the heart. Endogenous ACTA2 expression was observed in the cardiac region but in the brain region, which lacks smooth muscle cells (Fig. 2). These findings suggest that ACTA2 is endogenously expressed in the heart, as previously observed in the early development of the mammalian heart [9]. We subsequently evaluated cardiac function using Tg[*cmlc2*:EGFP] zebrafish, which were injected with high dose of *ACTA2* variants mRNA. The pathogenic variants of *ACTA2* have a dominant negative effect [7] and zebrafish animal model can theoretically be conducted by injecting human *ACTA2* pathogenic variants mRNA into fertilized zebrafish eggs. Following the fertilization of one-cell stage embryos, we injected in vitro synthesized *ACTA2* mRNA with the wild type and the novel variant G148R, along with the previously known pathogenic variant R179H as a positive control [5].

**Fig. 3:**
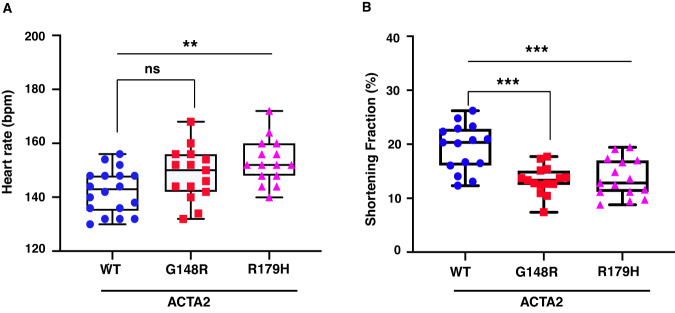
Heart contraction impairs in the *ACTA2* G148R variant. **A**, **B** Heart rate and shortening fraction measurement at 3 days post-fertilization. **A** Zebrafish with *ACTA2* p.G148R and *ACTA2* p.R179H show an increased heart rate (beats per minute). **B** Shortening fractions were significantly reduced in *ACTA2* G148R and *ACTA2* R179H. *P < 0.05, ***P < 0.001

The embryos were maintained and raised in E3 medium until 3 dpf for the heart analysis. No significant difference was observed in the body length between wild type and *ACTA2* pathogenic variants at 3 dpf (Supplementary Fig. S2). These results suggest that there was no growth retardation in the larvae zebrafish overexpressing *ACTA2* pathogenic variants. We then assessed cardiac contractility by analyzing the dynamic changing of signal intensity in EGFP fluorescence (Supplementary Fig. S1) [10]. While the heart rate of the R179H variant exhibited a significant increase compared to the wild type, the G148R variant did not show any significant difference in heart rate when compared to the wild type. However, the heart rate of the G148R variant tended to be higher when compared to the wild type (*P* = 0.0617) (Fig. 3A). In contrast, the SF was significantly reduced in both pathogenic variants (Fig. 3B). The observed tendency for higher heart rates in zebrafish with pathogenic variants may be a compensatory response to lower cardiac output, aimed at maintaining adequate circulating blood volume. This is consistent with the previous research that the heart rate was closely linked to cardiac output in zebrafish [12]. These results indicate that the G148R and R179H variants in *ACTA2* impair heart wall motion.

### ACTA2 pathogenic variants cause endocardial endothelium cells and cardiomyocytes cell defect in vivo

**Fig. 4:**
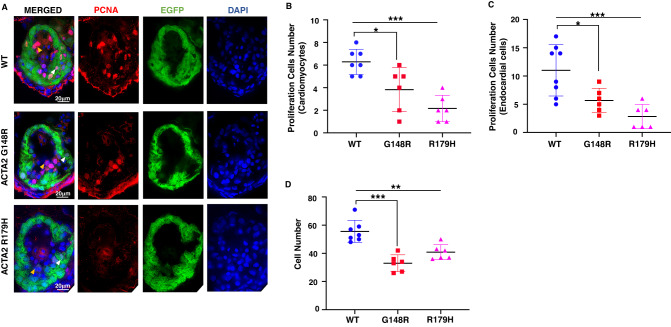
Proliferating cells were reduced in the *ACTA2* G148R variant. **A**–**D** Immunostaining of Tg[*cmlc2*:EGFP] *ACTA2* larvae heart at 4 days post-fertilization (green) with proliferation cell marker (PCNA, red), 4’,6-diamidino-2-phenylindole (DAPI) co-staining (blue). Scale bar: 20 μm. **A** Endocardial cells (yellow arrowheads) and cardiomyocytes (white arrowheads). Zebrafish with *ACTA2* p.G148R and *ACTA2* p.R179H show reduced total cell numbers. **B** In the myocardium, *ACTA2* p.G148R and *ACTA2* p.R179H cardiomyocyte showed decreased numbers of proliferating cells in the heart wall area highlighted with EGFP green fluorescence, PCNA, and DAPI. **C** In the endocardium area, endocardium cells showed decreased proliferation in all pathogenic variants highlighted with PCNA and DAPI. **P < 0.01, ***P < 0.001. Error bars indicate the standard deviation

To assess heart abnormalities in zebrafish with abundant mRNA of *ACTA2* pathogenic variants, we performed a histopathological evaluation. We counted proliferating cells (PCNA-positive cells) in the endothelium and myocardium. There were significantly fewer proliferating cells in the endothelial and myocardial regions of zebrafish with G148R or R179H than those in the wild type (Fig. 4A–C). In contrast, the proliferation cells were comparable between wild type and *ACTA2* pathogenic variants in the liver (Supplementary Fig. S3). The myocardial wall of zebrafish overexpressing *ACTA2* pathogenic variants was thinner than that of the wild type, and as a result of the decreased proliferating cells, we found the total cell numbers within the myocardium were apparently decreased in the zebrafish overexpressing the both pathogenic variants of *ACTA2* (Fig. 4D). The previous study demonstrated that aberrant proliferation of cardiomyocytes was detected in the developing hearts of zebrafish models with ventricular contractile defects [13]. Thus, histopathological characteristics demonstrated that the *ACTA2* pathogenic variants predominantly lead to the development of LVNC and cardiogenic defects.

## Discussion

This study identified a previously unreported heterozygous variant, G148R, in the *ACTA2* gene, manifesting in a patient with non-syndromic aortic aneurysm presenting with MSMD. Our findings in a zebrafish model demonstrate that this variant contributes to the pathophysiology of insufficient cardiomyocyte development, leading to impaired cardiac contractility. This further demonstrated that the distinctive phenotype of LVNC in humans is caused by a pathological variant of the *ACTA2* gene.

The zebrafish model is pivotal in understanding cardiac dysfunction caused by genetic variants with dominant negative effects. The zebrafish heart is structurally and functionally similar to that of humans, enabling noninvasive and comprehensive examinations of the cardiac function [14]. In the early developmental stage, the embryonic heart of zebrafish comprises only a few hundred cardiomyocytes, allowing for accurate and reproducible approaches to count the total number of cardiomyocytes in either or both chambers [14]. In this study, ACTA2 protein expression was localized to the myocardium and endocardium, and overexpression of the *ACTA2* G148R and R179H variants in the zebrafish model resulted in impaired heart contraction accompanied by specific inhibition of cell proliferation in both myocardial and endocardial endothelial cells. No abnormalities were observed in systemic tissues outside the heart of the larvae. It is plausible that zebrafish expressing ACTA2 variants represent the human phenotype of cardiac dysfunction associated with ACTA2 variants.

Less than 4% of heart failure patients are affected by the rare heart condition of LVNC, which is caused by abnormal trabeculation in the left ventricle [7]. The main pathological feature of LVNC is usually spongy myocardium. A previous study identified a causal role for cardiomyopathy in a cohort with a mutation in *PRDM16* and LVNC syndrome. Loss of *PRDM16* causes proliferative capacity defects during cardiogenesis in zebrafish [15]. In addition, abnormalities in the genes involved in the structural maintenance of cardiomyocytes are also considered to be the pathogenetic factor associated with LNVC disorder [16]. Studies have been conducted on non-compaction-like myocardium in animal models, showing alterations in the cell cycle of developing cardiomyocytes. A recent study using human-specific induced pluripotent stem cell-derived cardiomyocytes (iPSC-CMs) showed a defect in the development of cardiomyocytes associated with the pathogenesis of LVNC [17]. Thus, our data support these findings and suggest that a proliferation defect caused by a pathological human *ACTA2* G148R mutation contributes to the phenotypic features of LVNC.

The actin family is a well-known globular multifunctional protein that forms microfilaments. In vertebrates, α-actin polymerizes to form actin filaments and organize cytoskeleton, forming bundles or three-dimensional networks in skeletal, cardiac or smooth muscle cells. Amino acid residue G148 is located in subdomain 3 of ACTA2, which is crucial for the stability and polymerization of actin filaments, and near the hinge & lower hydrophobic cleft, predicted to interact with myosin [18, 19]. Previous reports have indicated that the amino acid residue G148 in ACTA2 can cause inherited aortic aneurysms and dissections, but the details were not well described. Another variant in this region, R149C, which is one amino acid residue towards the C-terminus, has also been reported to cause similar clinical presentation. Both variants located in subdomain 3 in the lower hydrophobic cleft, a site conserved in the actin family, suggesting their functional significance. The present case demonstrated MSDS and LVNC in addition to inherited aortic aneurysms and dissections. The cause of the phenotypic differences remains elusive, but the affected patients may exhibit a broad spectrum of clinical symptoms with varying degrees of severity. Further study is required to understand the correlation between clinical phenotypes and genetic variants.

Muscle contraction at the molecular level occurs through interactions between actin and myosin. Although ACTA2 is primarily expressed in SMCs [6–8], our results confirmed that ACTA2 abnormalities directly affect cardiomyocyte development. Each muscle type of skeletal, cardiac, and smooth possesses specific actin and myosin types. For instance, *ACTA1* encodes skeletal muscle α-actin, the main actin isoform in adult skeletal muscle that forms the core of the thin filament of the sarcomere [20]. Pathogenic variants in *ACTA1* cause congenital myopathy with a wide range of clinical variability, ranging from death in infancy to a survival in adulthood [21]. Furthermore, ACTN2, a cardiac-specific actin, is a well-known protein that primarily anchors and crosslinks actin filaments in the cardiac Z-disc at the lateral boundaries of the sarcomere, anchoring myofibrillar actin thin filaments and titin to Z-discs [22]. Previous studies have reported that an *ACTN2* mutation may lead to diverse cardiomyopathies, including cardiomyopathy, arrhythmia, and LVNC [22]. Certain pathological variants of *ACTN2* also influence the function and developmental process of skeletal muscles, causing congenital myopathy [22]. Thus, the gene expression of *ACTA2* or *ACTN2* is not strictly specific to smooth muscle and cardiomyocytes, respectively, but overlaps to some extent, which can explain the phenotypic overlaps observed between each genetic abnormality.

While the *ACTA2 gene* is primarily expressed in the smooth muscle of the large arteries, including the aorta, early detection of underlying genetic defects of the *ACTA2* gene is usually difficult because the extra-vascular symptoms are nonspecific [6]. However, our basic study confirmed that the effects of *ACTA2* abnormalities on cardiomyocytes accumulate to affect physiological functions, such as the heart rate and structure, in the zebrafish model. Consequently, we suspect that physiological cardiac tests and imaging examinations, such as ultrasound imaging, will prove useful for clinical screening of this disease. When ACTA2-related diseases are suspected by a genetic analysis, it is simple to evaluate the dominant negative effects of candidate variants in a relatively easy manner using a zebrafish model. In the future, this approach may be implemented in clinical practice, and drug screening using this model might contribute to disease prevention by identifying therapeutic agents. Further studies and repeated analyses using zebrafish are required in the future.

In conclusion, our study revealed that the *ACTA2* pathogenic variant was responsible for TAAD and LVNC. We gained a better understanding of the phenotypic features associated with ACTA2 pathogenesis using a zebrafish in vivo model. Our findings shed light on a broad range of clinical manifestations of genetic diseases in the actin family.

### Supplementary information

Supplementary Figures
