# Supplementary material for: Cardiac manifestations of human ACTA2 variants recapitulated in a zebrafish model
Source: J Hum Genet. 2024 Feb 5;69(3-4):133–8. doi: 10.1038/s10038-024-01221-0 (PMC10965439; doi:10.1038/s10038-024-01221-0)
Supplement: Supplementary file 1 — Supplementary Figures [file 10038_2024_1221_MOESM1_ESM.pptx]

## Slide 1
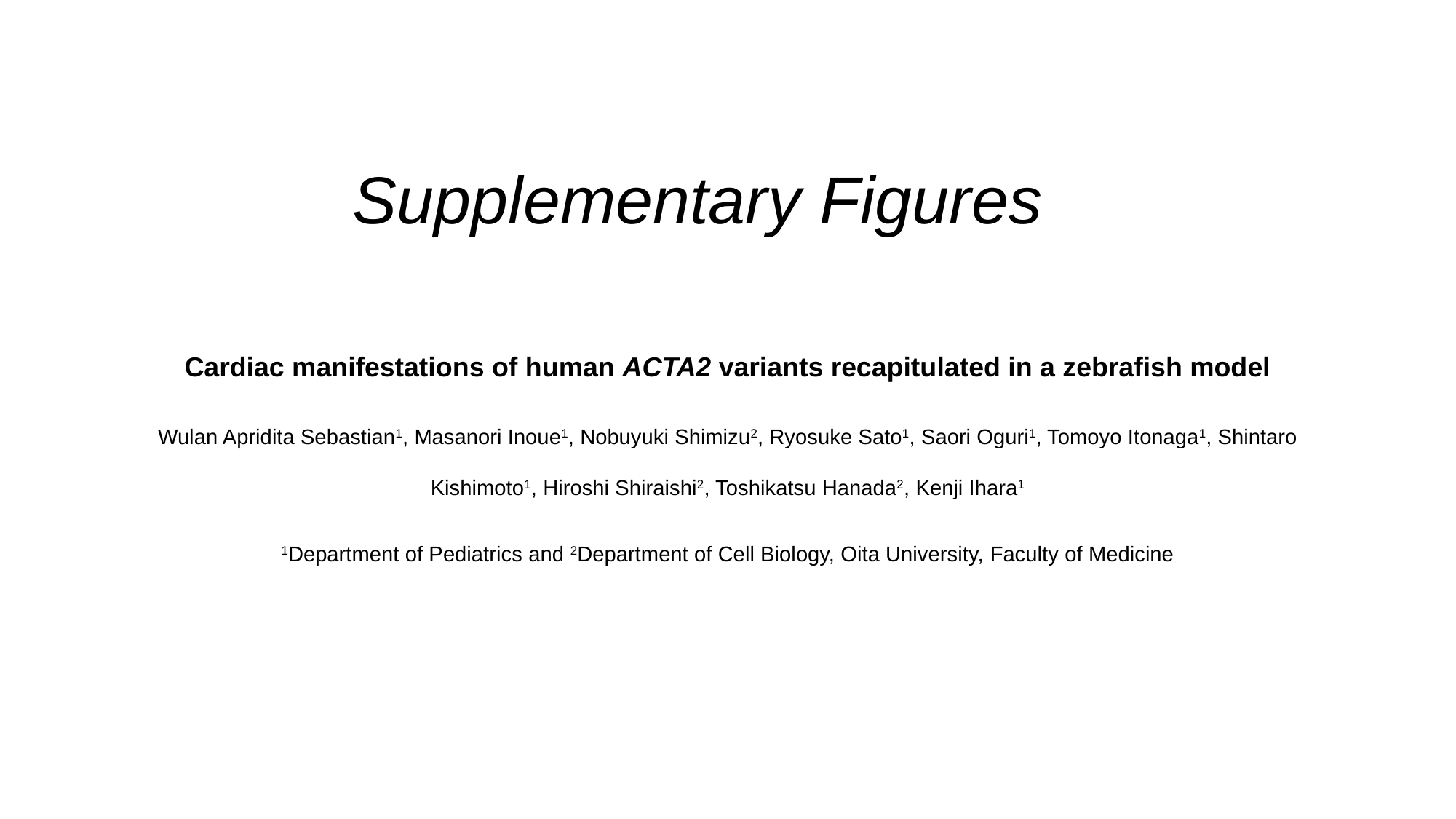

# Supplementary Figures
Cardiac manifestations of human ACTA2 variants recapitulated in a zebrafish model
Wulan Apridita Sebastian1, Masanori Inoue1, Nobuyuki Shimizu2, Ryosuke Sato1, Saori Oguri1, Tomoyo Itonaga1, Shintaro Kishimoto1, Hiroshi Shiraishi2, Toshikatsu Hanada2, Kenji Ihara1
1Department of Pediatrics and 2Department of Cell Biology, Oita University, Faculty of Medicine

## Slide 2
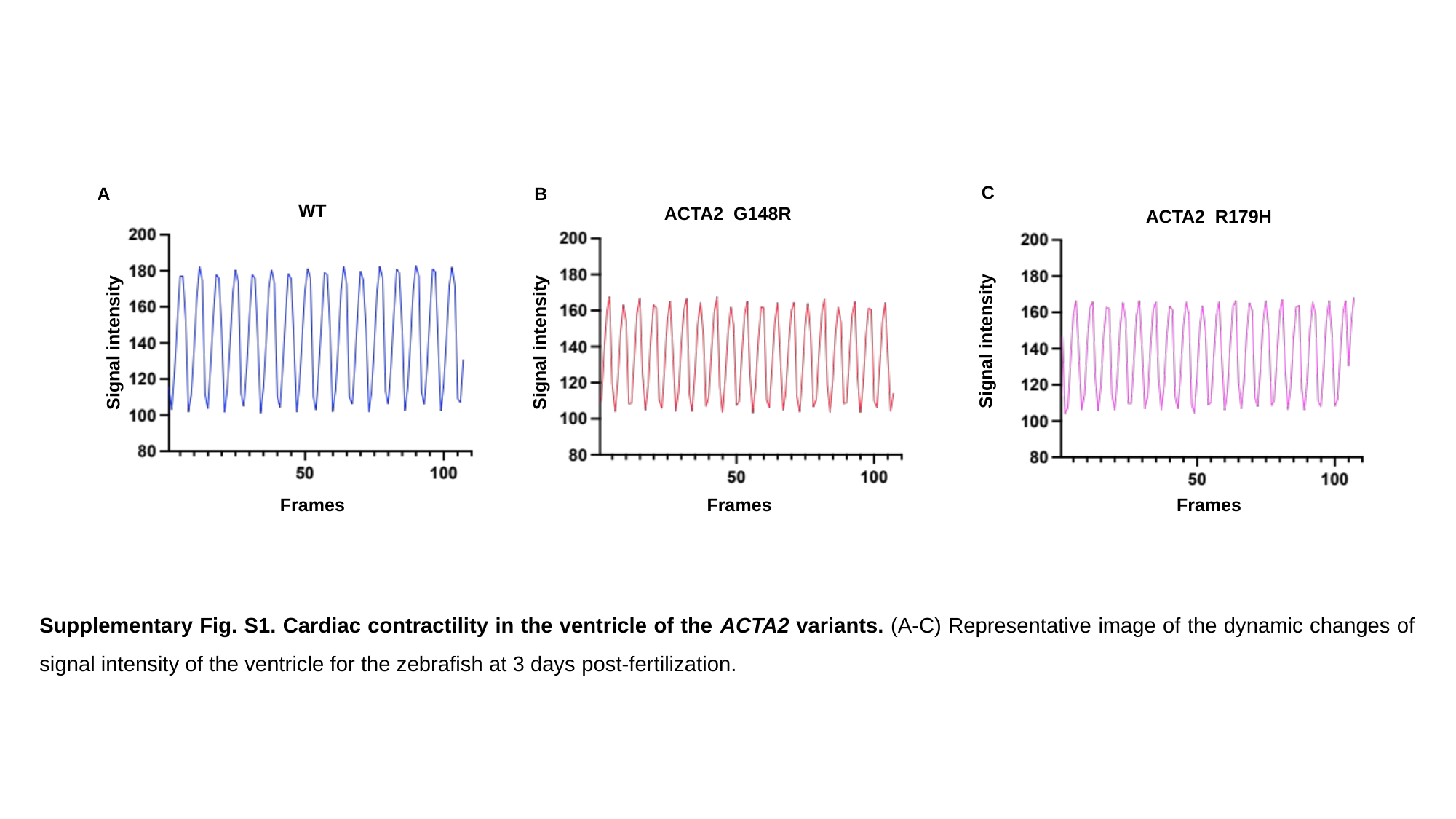

C
ACTA2 R179H
Signal intensity
Frames
A
WT
Signal intensity
Frames
B
ACTA2 G148R
Signal intensity
Frames
Supplementary Fig. S1. Cardiac contractility in the ventricle of the ACTA2 variants. (A-C) Representative image of the dynamic changes of signal intensity of the ventricle for the zebrafish at 3 days post-fertilization.

## Slide 3
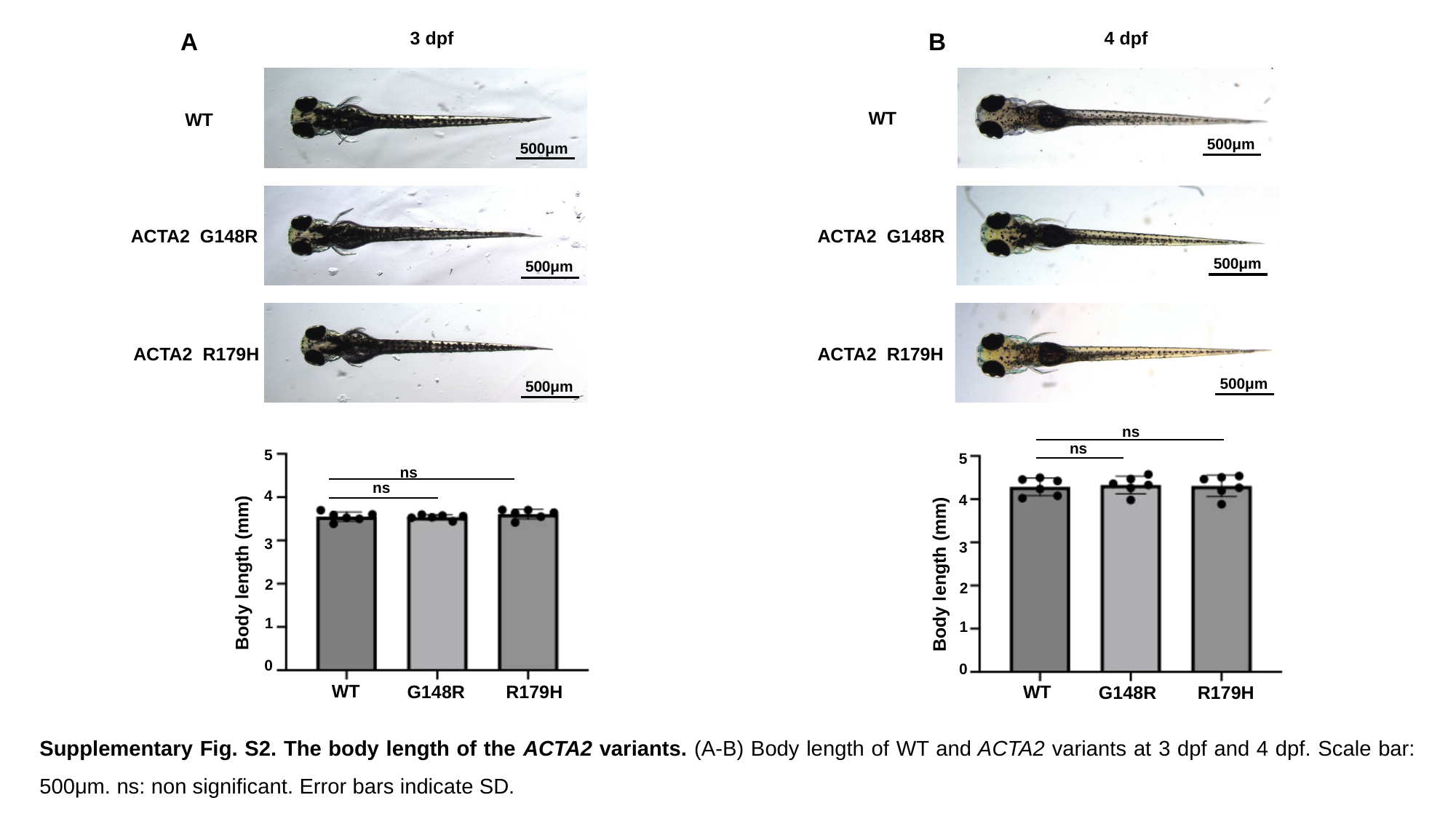

A
3 dpf
B
4 dpf
WT
WT
500μm
500μm
ACTA2 G148R
ACTA2 G148R
500μm
500μm
ACTA2 R179H
ACTA2 R179H
500μm
500μm
ns
ns
5
4
3
2
1
0
WT
R179H
G148R
5
4
3
2
1
0
ns
ns
WT
R179H
G148R
Body length (mm)
Body length (mm)
Supplementary Fig. S2. The body length of the ACTA2 variants. (A-B) Body length of WT and ACTA2 variants at 3 dpf and 4 dpf. Scale bar: 500μm. ns: non significant. Error bars indicate SD.

## Slide 4
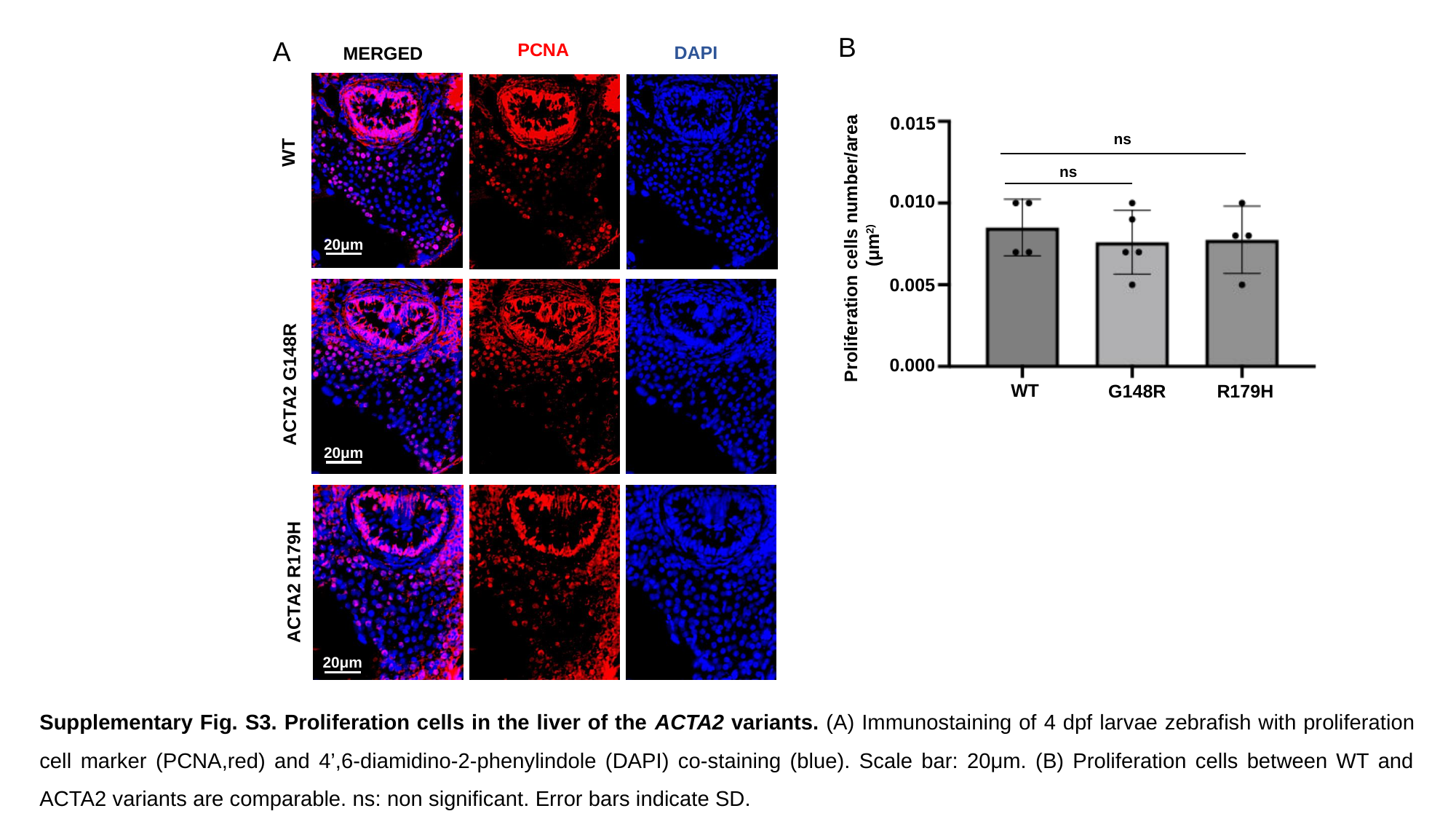

B
A
PCNA
DAPI
MERGED
WT
20μm
ACTA2 G148R
20μm
ACTA2 R179H
20μm
0.015
ns
ns
0.010
Proliferation cells number/area
(μm2)
0.005
0.000
WT
R179H
G148R
Supplementary Fig. S3. Proliferation cells in the liver of the ACTA2 variants. (A) Immunostaining of 4 dpf larvae zebrafish with proliferation cell marker (PCNA,red) and 4’,6-diamidino-2-phenylindole (DAPI) co-staining (blue). Scale bar: 20μm. (B) Proliferation cells between WT and ACTA2 variants are comparable. ns: non significant. Error bars indicate SD.
